# Establishment and application of a one-step multiplex real-time PCR assay for detection of A, B, and C subtypes of avian metapneumovirus

**DOI:** 10.1016/j.psj.2024.104608

**Published:** 2024-11-30

**Authors:** Mingjun Su, Jiongze Cheng, Xiangwen Xu, Yijia Liu, Yulin Zhao, Yutao Wang, Xiaoxu Du, Jiale Ying, Junfang Yan, Huihua Zheng, Changyong Cheng, Jing Sun

**Affiliations:** Key Laboratory of Applied Technology on Green-Eco-Healthy Animal Husbandry of Zhejiang Province, Zhejiang Provincial Engineering Research Center for Animal Health Diagnostics & Advanced Technology, Zhejiang International Science and Technology Cooperation Base for Veterinary Medicine and Health Management, China-Australia Joint Laboratory for Animal Health Big Data Analytics, College of Animal Science and Technology & College of Veterinary Medicine of Zhejiang A&F University, 666 Wusu Street, Lin'an District, Hangzhou, Zhejiang Province 311300, China

**Keywords:** Avian metapneumovirus, Multiplex real-time PCR assay, Receiver operating characteristic

## Abstract

Avian metapneumovirus (aMPV) represents a significant threat to the poultry industry, exhibiting a high degree of genetic diversity. Of these, the aMPV types A (aMPV-A), B (aMPV-B) and C (aMPV-C) are frequently detected in Chinese waterfowl and live poultry markets. Therefore, the rapid and accurate identification of these subtypes is of paramount importance in order to halt the spread of the disease. In this study, we have developed a multiplex real-time PCR assay endowed with the capacity to simultaneously discriminate aMPV-A, aMPV-B, and aMPV-C. This method demonstrates remarkable specificity, selectively amplifying aMPV-A, aMPV-B, and aMPV-C without cross-reactivity with other common avian pathogens. Furthermore, this method exhibits high sensitivity, with a detection threshold of 8.5 × 10^2^ copies/μL for aMPV-A, aMPV-B, and aMPV-C. Moreover, the assay demonstrates reproducibility, as evidenced by intra- and inter-assay variability, with a coefficient of variation between 0.21% and 1.91%. Additionally, the receiver operating characteristic (ROC) curve analysis demonstrated that the multiplex real-time PCR assay exhibited high specificity and sensitivity (100.0% and 100.0% for aMPV-A, 90.9% and 100.0% for aMPV-B, 100% and 96.8% for aMPV-C) when compared with the classical aMPV real-time RT-PCR. Analyses of field samples (n=105) using the multiplex real-time PCR assay indicated that 35.2% (37/105) of samples were positive for aMPV, of which 29.7% (11/37) for aMPV-A, 32.4% (12/37) for aMPV-B and 37.8% (14/37) for aMPV-C. These data demonstrated that the multiplex real-time PCR assay can be used for epidemiological investigations of tree subtypes of aMPV and that aMPV had been observed to exhibit a proclivity for multiple types of co-infection in the Zhejiang province of China.

## Introduction

Avian metapneumovirus (aMPV) poses a significant threat to the global poultry industry due to its propensity for causing acute respiratory infections among avian species (Graziosi et al., 2022a). aMPV, part of the *Paramyxoviridae* family, is characterized by a high degree of genetic diversity, which complicates efforts to control its spread (Afonso et al., 2016; Rima et al., 2017). In the poultry sector, aMPV infections can lead to severe economic repercussions, manifesting as drops in egg production, and increased mortality, which necessitates costly veterinary interventions and biosecurity measures (Cecchinato et al., 2018). Among the subtypes of aMPV, types A (aMPV-A), B (aMPV-B), and C (aMPV-C) are particularly noteworthy, especially in China where they have been frequently identified in waterfowl and live poultry markets (Bäyon-Auboyer et al., 2000; Juhasz and Easton, 1994; Seal, 2000; Wei et al., 2013). These settings serve as critical hubs for the virus, facilitating its transmission and genetic reassortment.

Given the rapid and often asymptomatic spread of aMPV, timely and accurate diagnosis of its subtypes is essential for effective disease management and containment (Kaboudi and Lachheb, 2021). Traditional diagnostic methods, such as serology and conventional PCR, are often limited by their turnaround time, sensitivity, and specificity, particularly when multiple subtypes are involved. The multiplex real-time PCR assay is highly specific and sensitive, accurately identifying different subtypes without cross-reactivity to other common avian pathogens (Zhang et al., 2019). This is important in live poultry markets where multiple pathogens may be present, and is also capable of detecting low-level infections that might otherwise be missed. Therefore, multiplex real-time PCR assay improves our ability to detect aMPV subtypes quickly and accurately, enhancing our understanding of the disease's spread and helping to prevent it, protecting poultry health and reducing economic losses.

This study describes the development of a multiplex real-time PCR assay capable of concurrently detecting and distinguishing between aMPV-A, aMPV-B, and aMPV-C. This novel assay represents combining the high-throughput capability with enhanced specificity and sensitivity. By employing specific molecular markers that are unique to each subtype, the assay ensures accurate subtype identification, a critical feature for epidemiological tracking and the deployment of subtype-specific interventions.

## Materials and methods

### Primers and probes

We analyzed all available aMPV-A, aMPV-B, and aMPV-C sequences in GenBank to improve primer detection efficiency. Subsequently, primers and probes were designed for detecting the conserved SH gene of aMPV-A and aMPV-B, as well as the P gene for aMPV-C. Using Vector NTI software, primers and probes were designed to correspond to highly conserved regions of the genome in these subtypes (Table 1). Probes were labeled with MGB at the 3′ end and three different fluorescent reporter dyes (FAM, CY5, HEX) at the 5′ end. This configuration allowed for simultaneous detection of aMPV subtypes A, B, and C in a single tube, leveraging the distinct emission wavelengths of the dyes. All primers and labeled TagMan probes were synthesized and modified by XiangYin (XY Biotechnology Co. Ltd, Hangzhou, China).

**Table 1:** Sequences of primers and probes sequences used in the multiplex real-time PCR assays

Primer or probe name	Target gene	Sequences (5′to3′)^a^	Nucleotide position	Size (bp)
aMPV-A-fwd	SH	TAGCTTTGATCTTCCTTGTTGC	5495 to 5516^b^	109
aMPV-A-rev	SH	GTAGTTGTGCTCGCTCCTGATA	5582 to 5603^b^	
aMPV-A-probe	SH	*FAM*-TCGGATTGTCTGTGAAAC-MGB	5522 to 5539^b^	
aMPV-B-fwd	SH	TAGTTTTGATCTTCCTTGTTGC	5499 to 5520^c^	109
aMPV-B-rev	SH	GTAGTTGTGCTCAGCTCTGATA	5586 to 5607^c^	
aMPV-B-probe	SH	*CY5*-CTGGCTGTCACAATAA-MGB	5543 to 5558^c^	
aMPV-C-fwd	P	GAGGATGCAAGCCGGTTGTA	1534 to 1553^d^	160
aMPV-C-rev	P	CTAGTGCTTCCATCTCAAGTTTTGTG	1668 to.1693^d^	
aMPV-C-probe	P	*HEX*-TTTGCTCCTACGAGTGATG-MGB	1564 to 1582^d^	

a FAM, 6-carboxyfluorescein; Cy5, cyanine 5; MGB, minor groove binding

b Based on the sequence of aMPV-A, strain IT/Ty/A/259-01/03 (GenBank accession number JF424833.1).

c Based on the sequence of aMPV-B, strain VCO3/60616 (GenBank accession number AB548428.1).

d Based on the sequence of aMPV-C, strain Colorado (GenBank accession number AY590688).

### Construction of plasmid standards

Specific target segments of aMPV-A, aMPV-B, aMPV-C, and full-length genes of SH and P of aMPV type D (aMPV-D) (Accession No: HG934339) were retrieved from the NCBI database (https://www.ncbi.nlm.nih.gov/) and synthesized. They were then cloned into the pUC57 vector by Genewiz Biotechnology (Suzhou, China). The plasmids of aMPV-A/B/C were mixed in a 1:1:1 ratio and diluted 10-fold in a gradient, each plasmid ranging from 8.5 × 10^6^ copies/μL to 8.5 × 10^1^ copies/μL, to serve as the standard plasmid.

### Optimization of primer and probe concentrations for multiplex real-time PCR assay

A series of experiments were conducted to optimize the multiplex real-time PCR method, utilizing various primer (0.50 µM, 0.75µM, and 1.00 µM) and probe (0.25 µM, 0.50 µM, and 0.75 µM) combinations. The plasmid standard, at a concentration of 8.5 × 10^4^ copies/μL, served as templates for the reaction. The multiplex real-time PCR assay was conducted on a CFX96 system (Bio-Rad) using a Superscript III Platinum One-Step Quantitative RT-PCR kit (Life Technologies, Carlsbad, California, USA), under the following conditions: 45°C for 30 min (reverse transcription), 95°C for 2 min (initial PCR activation step), followed by 50 cycles at 95°C 15 s (denaturation), 45°C for 30 s (annealing), and 72°C for 5 s (extension). The cycle threshold (Ct) values obtained from each experimental trial were collected and analyzed. Subsequently, the optimal working conditions were ascertained utilizing the MODDE 12.1 software.

### Sampling and RNA extraction

Samples positive for human metapneumovirus (hMPV), Newcastle disease virus (NDV), infectious bronchitis virus (IBV) and infectious laringotracheitis virus (ILTV) were stored at -80°C in our laboratory. Samples were treated with 3 to 5 volumes of PBS. Subsequently, the samples were vortexed and centrifuged at 12,000 × g at 4°C for 15 min to collect the supernatant. HiScript III All-in-one RT SuperMix Perfect for qPCR (Cat No: R333-01, Vazyme, Nanjing, China) was used for the reverse transcription reaction to obtain cDNA.

### Determination of specificity, sensitivity, and repeatability of the multiplex real-time PCR assay

To exclude potential false-positive results stemming from other viruses in the sample, a duplex real-time PCR detection method was used to detect aMPV-A, aMPV-B and aMPV-C standard plasmids, as well as the plasmids of aMPV SH and P gene, and other respiratory virus-positive samples preserved in our laboratory, including hMPV, NDV, IBV, and ILTV. RNase-free water served as a negative control. To assess the limit of detection of the duplex real-time PCR method, separate PCRs were conducted for both viruses using standard plasmid templates with concentrations ranging from 8.5 × 10^6^ to 8.5 × 10^1^ copies/μL. The conventional RT-PCR was performed using the Bio-Rad T100 system (Bio-Rad) according to the reaction system and procedure. The detection limits of real-time PCR and conventional RT-PCR were determined and compared for sensitivity. Multiplex real-time PCR was then performed using standard plasmids of aMPV-A, aMPV-B, and aMPV-C at concentrations ranging from 8.5 × 10^5^ copies/μL to 8.5 × 10^3^ copies/μL, with three replicates for each reaction. This experiment was repeated three times with a three-day interval between replicates. The same concentrations of aMPV-A, aMPV-B, and aMPV-C standard plasmids were mixed to form the detection template. To evaluate the repeatability, CV of Cq values was calculated for different concentrations of virus in samples based on the three tests.

### Validation of the multiplex real-time PCR assay

To assess the feasibility of the multiplex real-time PCR assay, A total 105 nasal turbinate samples were collected from commercial broiler flocks with significant clinical symptoms in Zhejiang Province between December 2017 to September 2018 (Table S1). These samples underwent testing using the multiplex real-time PCR assay. Each sample was tested three times, and the mean Ct value was used to determine positivity or negativity. Meanwhile, 105 samples were subjected to a real-time RT-PCR according to the protocol designed by Guionie et al. (2007) (Guionie et al., 2007) which is recommended by the Office International des Epizooties (OIE) (WOAH Terrestrial Manual, 2022, Chapter 3.3.15.- Turkey rhinotracheitis (avian metapneumovirus infections)). To evaluate the sensitivity and specificity of the multiplex real-time PCR assay, a receiver operating characteristic (ROC) curve was generated using the results of the nested-PCR as the standard for negative and positive determination. Statistical analysis was carried out by using SPSS software (Version 11.5 for windows, SPSS Inc., Chicago, IL, U.S.A.).

## Results

### Optimizing primer and probe concentrations for the multiplex reaction system

**Fig. 1:**
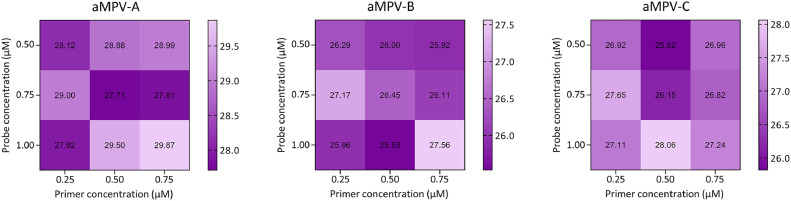
**Determination of the optimal concentration of primers and probes for Multiplex Real-Time PCR Assay.***Note*: The intensity of the color is inversely proportional to the Ct value.

To optimize amplification efficiency, multiplex real-time PCR was performed using probes at final concentrations ranging from 0.50 µM to 1.00 µM and primers at final concentrations ranging from 0.25 µM to 0.75 µM. Subsequently, the fluorescence intensity and Cq values of different combinations were analyzed. The results indicated that the most favorable outcomes were observed when utilizing a 0.5 µM primer and a 0.75 µM probe in a final volume of 10 µL (Fig. 1).

### Standard curve of the multiplex real-time PCR assay

**Fig. 2:**
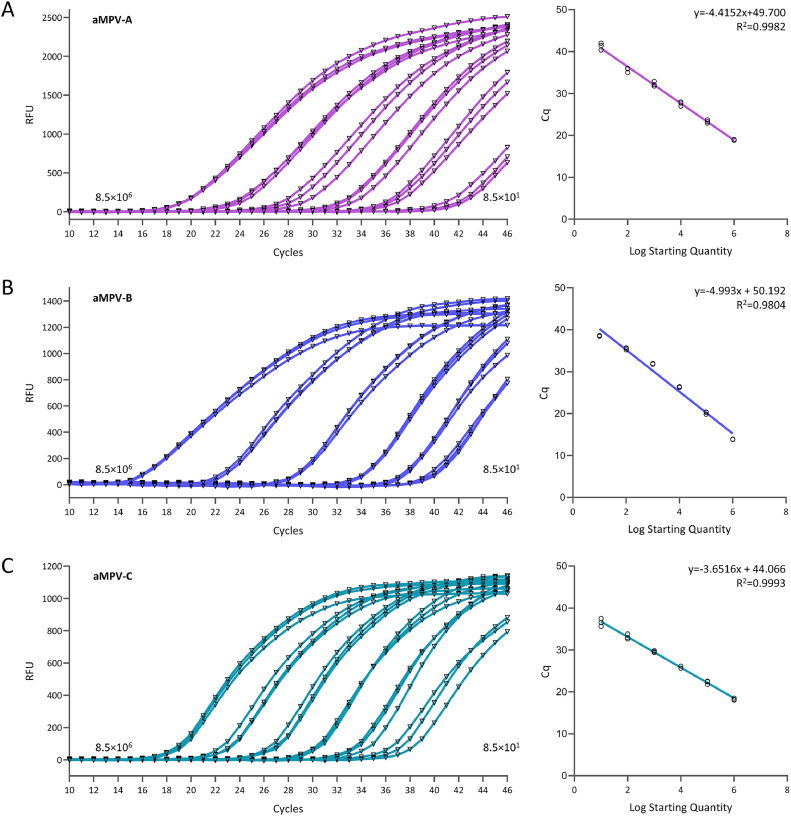
**Standard curves of the Multiplex Real-Time PCR Assay.** A-C. Standard curve different concentration of aMPV-1 (A), aMPV-2 (B), and aMPV-3 (C).

Plasmids of aMPV-A, aMPV-B, and aMPV-C were utilized as templates for the reaction, with concentrations ranging from 8.5 × 10^6^ copies/μL to 8.5 × 10^1^. The standard curves generated for aMPV-A, aMPV-B, and aMPV-C exhibited a linear relationship between the concentrations of the templates and their respective Ct values (Fig. 2). The equations derived from the linear regression analysis were as follows: Y = -4.4152x + 49.700 (R^2^ = 0.9982) for aMPV-A, Y = -4.993x + 50.192 (R^2^ = 0. 9804) for aMPV-B, and Y = -3.6516x + 44.066 (R^2^ = 0.9993) for aMPV-C. Above data demonstrated that the highly linear templates, across various dilutions, exhibited consistent and reproducible values, suggesting that the multiplex real-time PCR assay is both reliable and reproducible.

### Effectiveness of multiplex real-time PCR assay

**Fig. 3:**
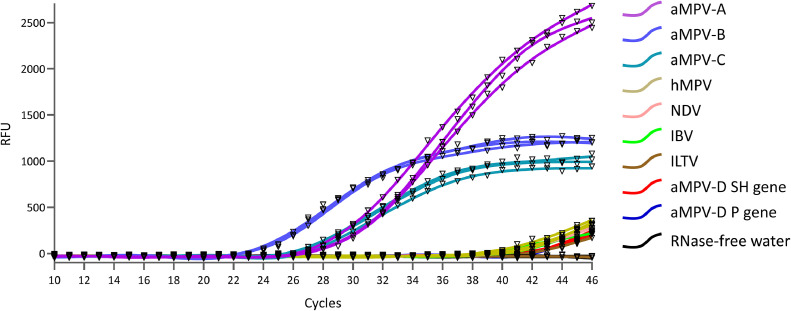
Specificity of the Multiplex Real-Time PCR Assay.

*Specificity:* In order to evaluate specificity, aMPV-A, aMPV-B, and aMPV-C were identified in samples positive for hMPV, NDV, IBV, ILTV, and the plasmids of SH gene and P gene of aMPV-D utilizing a refined methodology, with RNase-free water employed as a negative control. The results demonstrated that the method effectively identified the target pathogen while yielding negative results for all other viruses, suggesting that the method exhibits strong specificity in the specific detection of aMPV-A, aMPV-B, and aMPV-C in clinical samples (Fig. 3).

**Fig. 4:**
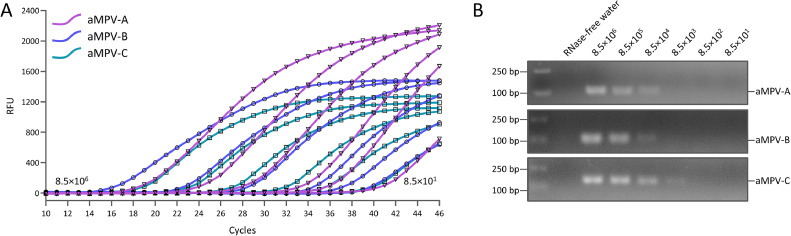
**Sensitivity of the Multiplex Real-Time PCR Assay.** A. Multiplex Real-Time PCR assay; B. Conventional PCR method.

*Sensitivity:* To evaluate the lower limit of detection of the multiplex real-time PCR assay, a range of dilutions (ranging from 8.5 × 10^6^ copies/μL to 8.5 × 10^1^ copies/μL) were utilized as templates for real-time PCR assays and conventional PCR in sensitivity testing. The results showed that the multiplex real-time PCR system detected positive standards at a minimum concentration of 8.5 × 10^2^ copies/μL, showing higher sensitivity than the conventional PCR method with a lower limit of detection of 8.5 × 10^4^ copies/μL for aMPV-A/B and 8.5 × 10^3^ copies/μL for aMPV-C (Fig. 4).

**Fig. 5:**
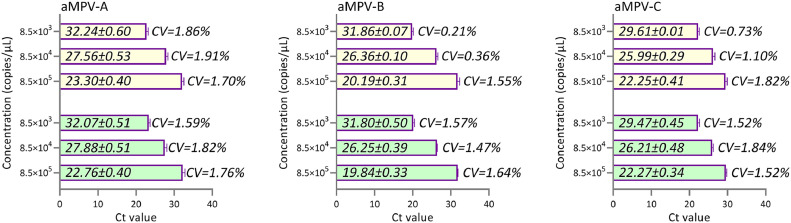
Intra-assay and inter-assay reproducibility of the Multiplex Real-Time PCR Assay.

*Repeatability:* Three replicates were conducted for repeatability tests, and coefficients of variation were calculated to analyze variability within and between assays. As demonstrated in Fig. 5, the coefficients of variation (CV) for the Cq values were consistently below 2.00% (with intra-assay CV ranging from 0.21% to 1.91% and inter-assay CV ranging from 1.74% to 1.84%), indicating that this method is stable and exhibits good repeatability.

### Validation of the multiplex real-time PCR assay

**Fig. 6:**
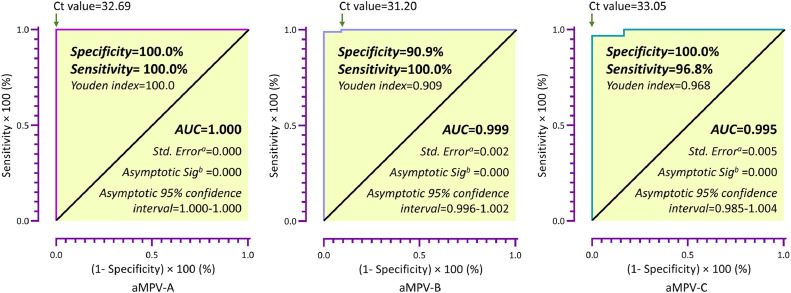
**The ROC curve using nested-RT PCR as diagnostic standard**. Note: a. Under the nonparametric assumption; b. Null hypothesis: true are=0.5.

**Fig. 7:**
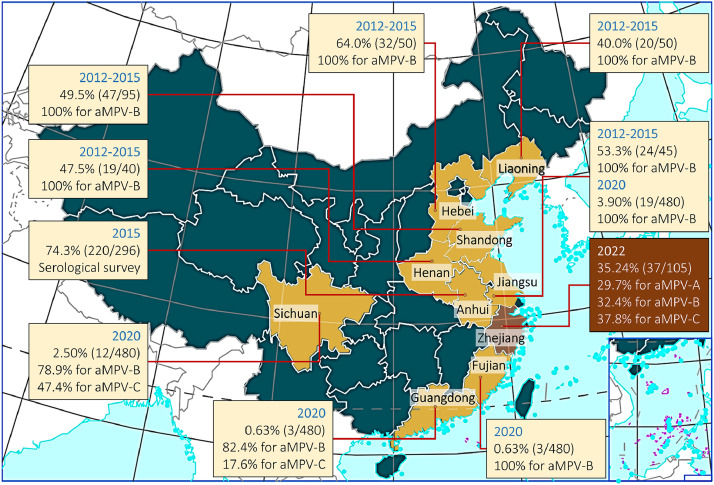
**The prevalence of aMPV-A, aMPV-B, and aMPV-C in China.** The base map was obtained from the Ministry of Natural Resources of China (http://bzdt.ch.mnr.gov.cn/).

In order to validate the multiplex real-time PCR assay, a total of 105 samples were tested using a real-time RT-PCR method described by Guionie et al. (2007) and published by OIE (Guionie et al., 2007). The ROC curve indicated that specificity and sensitivity of the multiplex real-time PCR assay were 100.0% and 100.0% for aMPV-A, 90.9% and 100.0% for aMPV-B, 100% and 96.8% for aMPV-C, and the area under ROC curve (AUC) were 1.000, 0.999, and 0.995 respectively (Fig. 6). Furthermore, the aforementioned aMPV-positive samples were detected into aMPV-A, aMPV-B and aMPV-C by the multiplex real-time PCR assay, resulting in positivity rates of 29.7% (11/37), 32.4% (12/37) and 37.8% (14/37), respectively. Analysis of published articles on the epidemiology of aMPV in China (selecting literature with complete sample information) showed that (Xiao et al., 2022; Zhang et al., 2017; Zhu et al., 2016), in the period between 2015 and 2020, aMPV was predominantly aMPV-B, followed by aMPV-C, and the data for 2022 demonstrated that aMPV-A, aMPV-B and aMPV-C were all prevalent genotypes (Table 2 and Fig. 7).

**Table 2:** Prevalence of aMPVs in China during 2012-2022.

Sampling Year	Sampling Location	Positive Rate				Type of Detection	Reference
		Total	aMPV-A	aMPV-B	aMPV-C		
2022	Zhejiang	35.24% (37/105)	29.7% (11/37)	32.4% (12/37)	37.8% (14/37)	Antigen	This study
2020	Fujian	0.63% (3/480)	0% (0/3)	100% (3/3)	0% (0/3)	Antigen	(Xiao et al., 2022)
	Jiangsu	2.50% (12/480)	0% (0/12)	100% (12/12)	0% (0/12)	Antigen	
	Guangdong	3.54% (17/480)	0% (0/17)	82.4% (14/17)	17.6% (3/17)	Antigen	
	Sichuan	3.96% (19/480) a	0% (0/19)	78.9% (15/19)	47.4% (9/19)	Antigen	
2015	Anhui	74.3% (220/296)	Unknown	Unknown	Unknown	Antibody	(Zhang et al., 2017)
2012-2015	Jiangsu	53.3% (24/45)	0% (0/24)	100% (24/24)	0% (0/24)	Antigen	(Zhu et al, 2016)
	Liaoning	40.0% (20/50)	0% (0/20)	100% (20/20)	0% (0/20)	Antigen	
	Henan	47.5% (19/40)	0% (0/19)	100% (19/19)	0% (0/19)	Antigen	
	Shandong	49.5% (47/95)	0% (0/47)	100% (47/47)	0% (0/47)	Antigen	
	Hebei	64.0% (32/50)	0% (0/32)	100% (32/32)	0% (0/32)	Antigen	

a Both aMPV-B and aMPV-C were detected in 5 aMPV-positive samples.

## Discussion

The findings of this study demonstrate the efficacy of the newly developed multiplex real-time PCR assay in the rapid and accurate detection of aMPV subtypes A, B, and C. This diagnostic tool addresses key challenges previously faced in the field, notably the need for high specificity and sensitivity in the detection of aMPV within mixed infections, which are common in live poultry markets. The assay's capacity to differentiate between the three subtypes without cross-reactivity with other avian pathogens overcoming the shortcomings of traditional methods that require separate testing for each subtype, as compared to conventional methods.

For diagnostic techniques, the assessment of sensitivity and specificity serves as crucial evaluation criteria. Presently, researchers have developed multiple real-time RT-PCR assays for the identification of aMPV antigen. Cecchinato et al. (2013) presented a real-time RT-PCR assay for the detection of aMPV-A and aMPV-B, and this assay demonstrated a detection limit of 10^-0.41^ TCID_50_/mL for aMPV-A and 10^1.15^ TCID_50_/mL for aMPV-B (Cecchinato et al., 2013). Lemaitre et al. (2022) demonstrated the efficacy of a digital droplet RT-PCR assay in distinguishing between the four aMPV subgroups, with an estimated limit of detection for extracted viral material ranging from 1 to 3 copies/µL (Lemaitre et al., 2022). Wang et al. (2022) developed a quadruple real-time RT-PCR assay for the detection of aMPVs, demonstrating a detection limit ranging from 10^2^ to 10^3^ cRNA copies per reaction, and the assay exhibited specificity by not cross-reacting with other prevalent poultry viruses, including avian influenza virus (AIV), NDV, IBV and ILTV (Wang et al., 2022). The multiplex real-time PCR assay established in this study showed high sensitivity, with a detection threshold of 10^1^ to 10^2^ copies/μL for aMPV-A, aMPV-B, and aMPV-C, and no cross-reactivity with hMPV, NDV, IBV and ILTV, which were in the similar order as the methods described by Wang et al. (2022). This heightened sensitivity and specificity facilitates the timely identification of aMPV infections, potentially preceding the manifestation of clinical symptoms, thereby enabling prompt intervention and containment of the virus. Early detection plays a critical role in live poultry markets characterized by rapid turnover and high bird density, which can promote the prevention of widespread virus dissemination. Furthermore, when compared to a classical aMPV real-time RT-PCR (Guionie et al., 2007), the method developed in this study demonstrated high sensitivity and specificity. This suggests that the established method exhibits high reliability and is suitable for detecting clinical samples.

The aMPV virus is widely distributed globally, exhibiting four distinct genotypes that vary in prevalence across different geographical regions (Graziosi et al., 2022a). In European countries aMPV has been reported in Greece, France, Italy and Tunisia, where aMPV-B is predominantly prevalent (Andreopoulou et al., 2019; Lachheb et al., 2023; Lupini et al., 2022) and aMPV-C is the dominant genotype in Italy (Graziosi et al., 2022b; Tucciarone et al., 2022); three genotypes are present in countries in the American region, with aMPV-A (Kariithi et al., 2022; Rivera-Benitez et al., 2014), aMPV-C (Jardine et al., 2018), and aMPV-B (Ongor et al., 2010) being the predominant prevalent genotypes in Mexican, Canadian, and United States avian populations, respectively. In addition, aMPV-B is the most prevalent strain in birds from Iran in the Middle East and Ethiopia in Africa (Hosseini et al., 2021; Mayahi et al., 2017; Motamed Chaboki et al., 2018). A limited number of reports have indicated that three aMPV genotypes, A, B, and C, exist in China. During the period between 2012 and 2015, the aMPV positivity rate in Jiangsu, Liaoning, Henan, Shandong, and Hebei provinces in China was 50.7%, and all of them were aMPV-B (Zhu et al., 2016); the aMPV positivity rate was 10.6% in samples collected in Fujian, Jiangsu, Guangdong, and Sichuan in 2020, with type B being the dominant genotype, followed by type C (Xiao et al., 2022). The findings of this research, which utilized multiplex real-time PCR to detect aMPV in poultry samples collected from Zhejiang Province in 2022, revealed a total positive rate of 35.24%, with type aMPV-A, aMPV-B, and aMPV-C accounting for 29.7%, 32.4%, and 37.8% respectively. These results suggest a high prevalence and genetic diversity of aMPV in China highlights the ongoing challenge of aMPV in commercial poultry operations and underscores the importance of robust surveillance systems. Consequently, the development of a multiplex real-time PCR assay capable of simultaneously identifying different aMPV genotypes is crucial for effective monitoring of the epidemic in China.

In conclusion, the multiplex real-time PCR assay developed in this study provides a significant improvement over existing diagnostic methods for aMPV by combining high specificity, sensitivity, and the ability to differentiate among key aMPV subtypes in a single test. Its implementation can greatly enhance the surveillance and control of aMPV in poultry populations, particularly in settings like live poultry markets that are critical points for disease transmission. Future research should focus on further validation of this assay across different regions and in various types of avian populations to fully understand its effectiveness and adaptability. Additionally, ongoing monitoring of the assay's performance and the genetic diversity of aMPV will be important to maintain its relevance and accuracy as the virus evolves.

## Author contributions

Jing Sun, and Mingjun Su conceived and designed the experiments. Mingjun Su, Jing Sun, Jiongze Cheng, Xiangwen Xu, Yulin Zhao, Yijia Liu, Yutao Wang, Xiaoxu Du, and Jiale Ying performed the experiments. Mingjun Su, Jiongze Cheng, Xiangwen Xu, Jing Sun, Junfang Yan, Huihua Zheng, and Changyong Cheng analyzed the data. Xiangwen Xu, Mingjun Su and Jing Sun wrote the manuscript. Mingjun Su, Jing Sun, Jiongze Cheng revised the manuscript. All authors have read and agreed to the published version of the manuscript.

Table S1. The source information of the serum sample.

## Disclosures

The authors declare that they have no competing interests.

## Appendix Supplementary materials

Image, application 1

## References

[bib0001] Afonso C.L., Amarasinghe G.K., Bányai K., Bào Y., Basler C.F., Bavari S., Bejerman N., Blasdell K.R., Briand F.X., Briese T., Bukreyev A., Calisher C.H., Chandran K., Chéng J., Clawson A.N., Collins P.L., Dietzgen R.G., Dolnik O., Domier L.L., Dürrwald R., Dye J.M., Easton A.J., Ebihara H., Farkas S.L., Freitas-Astúa J., Formenty P., Fouchier R.A., Fù Y., Ghedin E., Goodin M.M., Hewson R., Horie M., Hyndman T.H., Jiāng D., Kitajima E.W., Kobinger G.P., Kondo H., Kurath G., Lamb R.A., Lenardon S., Leroy E.M., Li C.X., Lin X.D., Liú L., Longdon B., Marton S., Maisner A., Mühlberger E., Netesov S.V., Nowotny N., Patterson J.L., Payne S.L., Paweska J.T., Randall R.E., Rima B.K., Rota P., Rubbenstroth D., Schwemmle M., Shi M., Smither S.J., Stenglein M.D., Stone D.M., Takada A., Terregino C., Tesh R.B., Tian J.H., Tomonaga K., Tordo N., Towner J.S., Vasilakis N., Verbeek M., Volchkov V.E., Wahl-Jensen V., Walsh J.A., Walker P.J., Wang D., Wang L.F., Wetzel T., Whitfield A.E., Xiè J.T., Yuen K.Y., Zhang Y.Z., Kuhn J.H. (2016). Taxonomy of the order Mononegavirales: update 2016. Arch. Virol..

[bib0002] Andreopoulou M., Franzo G., Tucciarone C.M., Prentza Z., Koutoulis K.C., Cecchinato M., Chaligianni I. (2019). Molecular epidemiology of infectious bronchitis virus and avian metapneumovirus in Greece. Poult. Sci..

[bib0003] Bäyon-Auboyer M.H., Arnauld C., Toquin D., Eterradossi N. (2000). Nucleotide sequences of the F, L and G protein genes of two non-A/non-B avian pneumoviruses (APV) reveal a novel APV subgroup. J. Gen. Virol..

[bib0004] Cecchinato M., Lupini C., Munoz Pogoreltseva O.S., Listorti V., Mondin A., Drigo M. (2013). Development of a real-time RT-PCR assay for the simultaneous identification, quantitation and differentiation of avian metapneumovirus subtypes A and B. Avian Pathol..

[bib0005] Cecchinato M., Lupini C., Silveira F., Listorti V., Catelli E. (2018). Molecular Characterization of Avian Metapneumovirus from Guinea Fowls (Numida meleagridis). Pakistan Veterinary Journal.

[bib0006] Graziosi G., Lupini C., Catelli E. (2022). Disentangling the role of wild birds in avian metapneumovirus (aMPV) epidemiology: A systematic review and meta-analysis. Transbound. Emerg. Dis..

[bib0007] Graziosi G., Mescolini G., Silveira F., Lupini C., Tucciarone C.M., Franzo G., Cecchinato M., Legnardi M., Gobbo F., Terregino C., Catelli E. (2022). First detection of avian metapneumovirus subtype C Eurasian lineage in a Eurasian wigeon (Mareca penelope) wintering in Northeastern Italy: an additional hint on the role of migrating birds in the viral epidemiology. Avian pathology: journal of the W.V.P.A.

[bib0008] Guionie O., Toquin D., Sellal E., Bouley S., Zwingelstein F., Allée C., Bougeard S., Lemière S., Eterradossi N. (2007). Laboratory evaluation of a quantitative real-time reverse transcription PCR assay for the detection and identification of the four subgroups of avian metapneumovirus. J. Virol. Methods.

[bib0009] Hosseini H., Ziafati Kafi Z., Malekan M., Ghafouri S.A., Fallah Mehrabadi M.H., Sadri N., Hojabr Rajeoni A., Ghalyanchilangeroudi A. (2021). Molecular characterization of circulating avian metapneumovirus, subgroup B, in broiler chickens, Iran, 2016-2018. Iran. J. Vet. Res..

[bib0010] Jardine C.M., Parmley E.J., Buchanan T., Nituch L., Ojkic D. (2018). Avian metapneumovirus subtype C in Wild Waterfowl in Ontario. Canada. Transboundary and emerging diseases.

[bib0011] Juhasz K., Easton A.J. (1994). Extensive sequence variation in the attachment (G) protein gene of avian pneumovirus: evidence for two distinct subgroups. J. Gen. Virol..

[bib0012] Kaboudi K., Lachheb J. (2021). Avian metapneumovirus infection in turkeys: a review on turkey rhinotracheitis. J. Appl. Poult. Res..

[bib0013] Kariithi H.M., Christy N., Decanini E.L., Lemiere S., Volkening J.D., Afonso C.L., Suarez D.L. (2022). Detection and Genome Sequence Analysis of Avian Metapneumovirus Subtype A Viruses Circulating in Commercial Chicken Flocks in Mexico. Vet. Sci..

[bib0014] Lachheb J., Bouslama Z., Nsiri J., Badr C., Al Gallas N., Souissi N., Khazri I., Larbi I., Kaboudi K., Ghram A. (2023). Phylogenetic and phylodynamic analyses of subtype-B metapneumovirus from chickens in Tunisia. Poult. Sci..

[bib0015] Lemaitre E., Bougeard S., Allée C., Eterradossi N., Courtillon C., Brown P.A. (2022). Avian metapneumovirus: A five-plex digital droplet RT-PCR method for identification of subgroups A, B, C, and D. Front. Vet. Sci..

[bib0016] Lupini C., Tucciarone C.M., Mescolini G., Quaglia G., Graziosi G., Turblin V., Brown P., Cecchinato M., Legnardi M., Delquigny T., Lemiere S., Perreul G., Catelli E. (2022). Longitudinal Survey on aMPV Circulation in French Broiler Flocks following Different Vaccination Strategies. . Animals: an open access journal from MDPI.

[bib0017] Mayahi M., Momtaz H., Jafari R.A., Zamani P. (2017). Detection and subtyping avian metapneumovirus from turkeys in Iran. Veterinary research forum: an international quarterly journal.

[bib0018] Motamed Chaboki P., Ghalyanchilangeroudi A., Karimi V., Abdollahi H., Maghsoudloo H., Hosseini H., Khaltababdi Farahahni R., Ghafouri S.A., Falah M.H., Rezaee H., Jabbarifakhr M., Mousavi F.S. (2018). Prevalence of avian metapneumovirus subtype B in live bird market in Gilan province. Iran. Veterinary research forum: an international quarterly journal.

[bib0019] Ongor H., Karahan M., Kalin R., Bulut H., Cetinkaya B. (2010). Detection of avian metapneumovirus subtypes in turkeys using RT-PCR. Vet. Rec..

[bib0020] Rima B., Collins P., Easton A., Fouchier R., Kurath G., Lamb R.A., Lee B., Maisner A., Rota P., Wang L., Report C.Ictv (2017). ICTV Virus Taxonomy Profile: Pneumoviridae. J. Gen. Virol..

[bib0021] Rivera-Benitez J.F., Martínez-Bautista R., Ríos-Cambre F., Ramírez-Mendoza H. (2014). Molecular detection and isolation of avian metapneumovirus in Mexico. Avian pathology: journal of the W.V.P.A.

[bib0022] Seal B.S. (2000). Avian pneumoviruses and emergence of a new type in the United States of America. Anim. Health Res. Rev..

[bib0023] Tucciarone C.M., Franzo G., Legnardi M., Pasotto D., Lupini C., Catelli E., Quaglia G., Graziosi G., Dal Molin E., Gobbo F., Cecchinato M. (2022). Molecular Survey on A, B, C and New Avian Metapneumovirus (aMPV) Subtypes in Wild Birds of Northern-Central Italy. Vet. Sci..

[bib0024] Wang S., Jiang N., Jiang L., Zhuang Q., Chen Q., Hou G., Xiao Z., Zhao R., Li Y., Zhao C., Zhang F., Yu J., Li J., Liu H., Sun F., Wang K. (2022). Establishment and application of a quadruple real-time RT-PCR for detecting avian metapneumovirus. PLoS. One.

[bib0025] Wei L., Zhu S., Yan X., Wang J., Zhang C., Liu S., She R., Hu F., Quan R., Liu J. (2013). Avian metapneumovirus subgroup C infection in chickens. Emerg. Infect. Dis..

[bib0026] Xiao Z., Wang S., Zhao C., Huang Y., Chen H., Cui C., Sun F., Wang K. (2022). Epidemiological investigation of avian metapneumovirus in some areas of China in 2020. Chin. J. Prevent. Veterinar. Med..

[bib0027] Zhang D., Dai Y., Zhao R., Hu X., Shen X., Hou H., Pan X., Zhou X., Zhu C. (2017). Serological survey on avian metapneumovirus infection in chicken flocks in Anhui province. Proger. Veterinar. Med..

[bib0028] Zhang Z., Liu D., Hu J., Sun W., Liu K., Li J., Xu H., Liu J., He L., Jiang D., Gu M., Hu S., Wang X., Liu X., Liu X. (2019). Multiplex one-step real-time PCR assay for rapid simultaneous detection of velogenic and mesogenic Newcastle disease virus and H5-subtype avian influenza virus. Arch. Virol..

[bib0029] Zhu Y., Gong X., Guo W., Xu B., Li L., Lang F., Liu H., Liu D., Fan G. (2016). Molecular epidemiology analysis of aMPV in some regions in China during 2012 to 2015. Proger. Veterinar. Med..

